# Is Occult Obscure Gastrointestinal Bleeding a Definite Indication for Capsule Endoscopy? A Retrospective Analysis of Diagnostic Yield in Patients with Occult versus Overt Bleeding

**DOI:** 10.1155/2013/915463

**Published:** 2013-11-12

**Authors:** Ikue Watari, Shiro Oka, Shinji Tanaka, Makoto Nakano, Taiki Aoyama, Shigeto Yoshida, Kazuaki Chayama

**Affiliations:** ^1^Department of Gastroenterology and Metabolism, Graduate School of Biomedical & Health Sciences, Hiroshima University, 1-2-3 Kasumi, Minami-ku, Hiroshima 734-8551, Japan; ^2^Department of Endoscopy, Hiroshima University Hospital, 1-2-3 Kasumi, Minami-ku, Hiroshima 734-8551, Japan

## Abstract

*Background/Aim*. Usefulness of capsule endoscopy (CE) for diagnosing small-bowel lesions in patients with obscure gastrointestinal bleeding (OGIB) has been reported. Most reports have addressed the clinical features of overt OGIB, with few addressing occult OGIB. We aimed to clarify whether occult OGIB is a definite indication for CE. *Methods*. We retrospectively compared the cases of 102 patients with occult OGIB and 325 patients with overt OGIB, all having undergone CE. The diagnostic yield of CE and identification of various lesion types were determined in cases of occult OGIB versus overt OGIB. *Results*. There was no significant difference in diagnostic yield between occult and overt OGIB. The small-bowel lesions in cases of occult OGIB were diagnosed as ulcer/erosive lesions (*n* = 18, 18%), vascular lesions (*n* = 11, 11%), and tumors (*n* = 4, 3%), and those in cases of overt OGIB were diagnosed as ulcer/erosive lesions (*n* = 51, 16%), vascular lesions (*n* = 31, 10%), and tumors (*n* = 20, 6%). *Conclusion*. CE detection rates and CE identification of various small-bowel diseases do not differ between patients with occult versus overt OGIB. CE should be actively performed for patients with either occult or overt OGIB.

## 1. Introduction

Obscure gastrointestinal bleeding (OGIB) accounts for approximately 5% of all cases of GI bleeding and is frequently due to a lesion in the small bowel [1–4]. OGIB is defined as bleeding from the gastrointestinal tract that persists or recurs without an obvious source being discovered by esophagogastroduodenoscopy (EGD), colonoscopy, or radiologic evaluation of the small bowel, that is, small bowel follow-through (SBFT) or enteroclysis [3]. OGIB is classified as either occult or overt, with occult OGIB defined as iron deficiency anemia (IDA), with or without a positive fecal occult blood test [5, 6], and overt OGIB defined as clinically perceptible bleeding that recurs or persists despite negative initial endoscopic (EGD and colonoscopy) and radiologic evaluations (SBFT or enteroclysis).

Usefulness of capsule endoscopy (CE) for diagnosing small-bowel lesions in patients with OGIB has been reported [7–10]. CE is used especially in patients with overt OGIB, with most previous reports addressing mainly the clinical features of overt OGIB. Few reports address the clinical features of occult OGIB. We conducted a comparative retrospective study to clarify whether occult OGIB is a definite indication for CE.

## 2. Material and Methods

### 2.1. Patients

From our hospital records, we identified 427 patients who had undergone CE for OGIB between April 2006 and February 2013: 102 with occult OGIB and 325 with overt OGIB. Patients with ongoing overt OGIB were not included in this study.

Occult OGIB was defined as IDA and/or a positive fecal occult blood test, and overt OGIB was defined as clinically perceptible bleeding that recurs or persists despite negative initial endoscopic (EGD and colonoscopy) and radiologic evaluations (SBFT or enteroclysis).

IDA was diagnosed according to standard criteria, that is, a blood hemoglobin concentration of <13.8 g/dL for men, <11.5 g/dL for postmenopausal women, and <11 g/dL for premenopausal women, with a plasma ferritin level of <30 mg/L and a mean corpuscular volume of <80 fL [11]. Occult blood in the stool was detected by immunochemical fecal occult blood test. Transabdominal ultrasonography (TUS) and/or abdominal computed tomography (CT) were performed to uncover stenosis of the gastrointestinal tract and/or small-bowel disease before CE in all patients [12].

### 2.2. CE Examinations and Findings

When CE was performed, the CE capsule (PillCam SB1/SB2; Given Imaging Ltd., Yoqneam, Israel) was swallowed with a solution of dimethicone after an overnight fast. Most patients were given 34 g magnesium citrate for bowel preparation on the night before the procedure. Patients were allowed to drink clear liquids 2 hours after swallowing the capsule and to eat a light meal at 4 hours. Images were analyzed with Rapid Reader 4.0/5.0/6.5 software on a RAPID workstation (both from Given Imaging). CE images were reviewed independently by two gastroenterologists. If the gastroenterologists' findings differed, consensus was reached through discussion. Total CE was considered successful when the capsule reached the cecum within the recording time. Capsule retention was defined as a capsule remaining in the digestive tract for a minimum of 2 weeks.

CE findings were categorized as positive when a bleeding source was detected within the small bowel and as negative when no bleeding source was detected within the small bowel. We defined a bleeding source as a lesion with obvious bleeding (active bleeding or blood clot) or a lesion without obvious bleeding but that could be the cause of bleeding. Some detected lesions were considered not to be sources of bleeding, such as small red spots and erosions without active bleeding or blood clot. Small-bowel lesions were subclassified as vascular, ulcer/erosion, tumor, or other types of lesion.

### 2.3. Data Collection

Patients' clinical records were obtained, and demographic, clinical, procedural, and diagnostic data were extracted for analyses. Information gathered included age, sex, type of gastrointestinal bleeding (occult versus overt), hemoglobin concentration upon CE examination, plasma ferritin level upon CE examination, need for blood transfusion, time from the first OGIB episode, previous endoscopic diagnosis, CE findings, and results of pathologic examination of biopsy specimens obtained by double balloon endoscopy (DBE) or surgery. The total CE rate and the CE complication rate were determined for each of the 2 study groups (occult OGIB and overt OGIB). The diagnostic yield was determined in each group in terms of the detection of small-bowel lesions and identification of the various types of small-bowel lesions. In cases of occult OGIB, patient characteristics were examined in relation to lesion types.

Continuous data are presented as mean ± SD, and categorical data are presented as frequencies (percentages). Between-group differences in age and laboratory values were analyzed by Student's *t*-test. The proportions of patients with small-bowel lesions and no small-bowel lesions were compared by Fisher's exact test. The proportions of patients with vascular lesions, ulcer or erosion, and tumor were also analyzed by Fisher's exact test. All analyses were performed with JMP-J software. *P* < 0.05 was considered statistically significant.

The study protocol was approved by the ethics committee of Hiroshima University Hospital, and the study was conducted in accordance with the principles of the Declaration of Helsinki.

## 3. Results

Patient characteristics are shown per study group (occult OGIB and overt OGIB) in Table 1. There was no significant difference between the 2 groups in age, time from the first OGIB episode, or hemoglobin concentration at the time of examination. The plasma ferritin level was significantly lower in the occult OGIB group than in the overt OGIB group (*P* = 0.0003). Moreover, the percentage of patients requiring blood transfusion was significantly lower in the occult OGIB group (16%, *n* = 16) than in the overt OGIB group (34%, *n* = 110) (*P* < 0.01). Indications for CE among patients with occult OGIB were recurrent/persistent IDA (*n* = 68), recurrent/persistent IDA in addition to a positive fecal occult blood test (*n* = 29), and a positive fecal occult blood test (*n* = 5).

Total CE was achieved in 74 of the 102 patients (73%) with occult OGIB and in 234 of the 325 patients (72%) with overt OGIB, with no significant difference in the total CE rate between the 2 groups (*P* = 0.91). Capsule retention was noted in 1 of the 102 patients (1%) with occult OGIB and in 2 of the 325 patients (0.6%) with overt OGIB. The occult OGIB patient was found to have small-bowel strictures resulting from tuberculosis. One of the 2 overt OGIB patients had a strictured small bowel resulting from tuberculosis, and the other had a nonspecific ulcer. Two of these 3 patients underwent DBE and 1 underwent surgery to remove the capsule.

The final diagnoses are shown in Table 2. Among the occult OGIB patients, the final diagnosis was either small-bowel lesion(s) (*n* = 33, 32%) or no lesion in the small-bowel (*n* = 69, 68%). Among the overt OGIB patients also, the final diagnosis was either small-bowel lesion(s) (*n* = 106, 33%) or no lesion in the small bowel (*n* = 219, 67%), with no significant difference in diagnostic yield between the 2 groups. Among patients with occult OGIB, small-bowel lesions were ulcer or erosive lesion(s) (*n* = 18, 18%), vascular lesion(s) (*n* = 11, 11%), and tumor(s) (*n* = 4, 3%), whereas lesions among overt OGIB patients were ulcer or erosive lesion(s) (*n* = 51, 16%), vascular lesions (*n* = 31, 10%), and tumor(s) (*n* = 20, 6%), with no difference in the various small-bowel lesion types between the 2 groups.

Of the 18 ulcer/erosive lesions found in patients with occult OGIB, NSAID ulcer was most common (*n* = 10, 55%), followed by non-specific ulcer/erosion (*n* = 3, 16%), intestinal tuberculosis (*n* = 2, 11%), Crohn's disease (*n* = 1, 6%), chronic nonspecific multiple ulcers of the small intestine (CNSU) (*n* = 1, 6%), and radiation enterocolitis (*n* = 1, 6%). Of the 51 ulcer/erosive lesions found in patients with overt OGIB, nonspecific ulcer/erosion was most common (*n* = 18, 35%), followed by NSAID ulcer (*n* = 13, 25%), anastomotic ulcer (*n* = 6, 12%), intestinal tuberculosis (*n* = 4, 8%), Crohn's disease (*n* = 3, 6%), Henoch-Schönlein purpura (*n* = 2, 4%), Behçet's disease (*n* = 1, 2%), radiation enterocolitis (*n* = 1, 2%), CNSU (*n* = 1, 2%), eosinophilic gastroenteritis (*n* = 1, 2%), and amyloidosis (*n* = 1, 2%). There was no celiac disease.

Of the 11 vascular lesions found in patients with occult OGIB, angioectasia was most common (*n* = 9, 82%), followed by hemangioma (*n* = 1, 9%) and blue rubber bleb nevus syndrome (*n* = 1, 9%). Of the 31 vascular lesions found in patients with overt OGIB, angioectasia was most common (*n* = 23, 74%), followed by hemangioma (*n* = 4, 13%), arteriovenous malformation (*n* = 3, 10%), and varices (*n* = 1, 3%).

Characteristics of the 4 cases of occult OGIB arising from a tumor are given in Table 3. Tumor types were as follows: jejunal carcinoma (*n* = 1, 25%), gastrointestinal stromal tumor (GIST) (*n* = 1, 25%), aberrant pancreas (*n* = 1, 25%), and T-cell lymphoma (*n* = 1, 25%). Of the 20 tumors identified in patients with overt OGIB, GIST was the most common (*n* = 9, 45%), followed by adenoma/hamartomatous polyp(s) (*n* = 3, 15%), lipoma (*n* = 2, 10%), ectopic gastric mucosa (*n* = 1, 5%), carcinoid tumor (*n* = 1, 5%), primary small-bowel cancer (*n* = 1, 5%), aberrant pancreas (*n* = 1, 5%), malignant lymphoma (*n* = 1, 5%), and polyp in a case of familial adenomatous polyposis (FAP) (*n* = 1, 5%).

Diagnostic (final) and treatment modalities are shown per bleeding type in Table 4. Among patients with occult OGIB, vascular lesions were diagnosed endoscopically, that is, on the basis of CE and/or DBE findings (*n* = 11). Ulcer or erosive lesions were diagnosed endoscopically on the basis of CE and/or DBE findings (*n* = 14), response to medical treatment (*n* = 3), or DBE biopsy (*n* = 1). Tumors were diagnosed by surgery (*n* = 3) or DBE biopsy (*n* = 1). Among patients with overt OGIB, vascular lesions were diagnosed endoscopically on the basis of CE and/or DBE findings (*n* = 31). Ulcer/erosive lesions were diagnosed endoscopically on the basis of CE and/or DBE findings (*n* = 48), DBE biopsy (*n* = 2), or response to medical treatment (*n* = 1). Tumors were diagnosed upon surgery (*n* = 12), upon resection under DBE (*n* = 4), endoscopically on the basis of CE findings (*n* = 3), or by DBE biopsy (*n* = 1). Six of the 11 occult OGIB patients (55%) with a vascular lesion underwent endoscopic hemostasis, 7 of the 18 patients (39%) with ulcer/erosive lesion received treatment, and the 4 patients (100%) with tumor underwent surgery or chemotherapy. Of patients with overt OGIB, 27 of the 31 patients (87%) with a vascular lesion underwent endoscopic hemostasis or interventional radiology. Thirty of the 51 patients (59%) with an ulcer or erosive lesion received treatment. Seventeen of the 20 patients (85%) with tumor underwent surgery, endoscopic resection, or chemotherapy.

Clinical characteristics of patients with occult OGIB are shown per positive and negative CE examinations in Table 5. The hemoglobin concentration was significantly high in patients in whom an ulcer or erosive lesion was found in comparison to the concentration in patients in whom no small-bowel lesion was found. The platelet count was significantly high in patients in whom a tumor was found in comparison to that in patients in whom no small-bowel lesion was found. The white blood cell count, hemoglobin concentration, platelet count, and total protein, serum albumin, CRP, and plasma ferritin levels did not differ between patients in whom a vascular lesion was found and those in whom no small-bowel lesion was found.

## 4. Discussion

The reported overall diagnostic yield of CE [13] and/or balloon endoscopy [14, 15] in patients with OGIB is 46–81% [8, 9, 16–30]. In cases of occult OGIB, specifically, the diagnostic yield of DBE is 42.1–76.5% [16, 24] and CE is 39–64.1% [9, 25–29]. In cases of overt OGIB, specifically, the diagnostic yield of CE or DBE is 53–87% [9, 17, 24, 25, 27–30]. Although DBE allows for tissue biopsy, small-bowel lesions are detected at the same rate by CE and DBE [31–33]. A diagnostic yield of 32% was achieved among our patients with occult OGIB due to small-bowel lesions, and this yield was similar to yields previously reported [9, 16, 24–29]. Sun et al. [24] reported an even higher diagnostic yield of 76.5% with DBE in patients with occult OGIB. We believe the difference in yield between their study and ours was due to the fact that their count included lesions outside the small intestine.

IDA is one of the major symptoms in patients with occult OGIB. Yamada et al. [34] performed CE in patients with IDA but without abnormalities found upon upper and lower endoscopy and reported that clinically significant lesions were statistically more prevalent in patients with IDA than in healthy volunteers (46% versus 15%).

Apostolopoulos et al. [35] performed CE in patients with IDA but without upper and lower endoscopic abnormalities and reported discovery of small-bowel lesions in 57% of cases. These data, together with the results of our study, lead us to believe that small-bowel examination is necessary in patients with IDA.

The diagnostic yield in our patients with overt OGIB was low compared to yields previously reported [9, 17, 24, 25, 28–30]. Patients with ongoing OGIB were included in these reported studies. The previously reported diagnostic yield among patients with ongoing OGIB is 76–92% [36–39], suggesting that features of ongoing overt OGIB differ from those of previous overt OGIB. We think that the discrepancy between previously reported diagnostic yields and our diagnostic yield may be due to our exclusion of ongoing OGIB. Investigation regarding any relation between the time to examination (time between presentation and examination) and the diagnostic yield is difficult in cases of occult OGIB; however, we believe that the diagnostic yield may differ according to the time between detection of fecal occult blood and/or diagnosis of chronic anemia and the time of small-bowel endoscopy. Future analysis of this issue is needed.

Ulcer or erosive lesion was the most common small-bowel lesion in our patients, whether those with occult OGIB or those with overt OGIB. In a fairly recent study, tumor was the most frequent source of bleeding in patients with either type of OGIB [24]. In a second fairly recent study, vascular lesions were the most frequent source of bleeding regardless of the type of OGIB [28], and in another study, ulcer/erosion was the most frequent source of bleeding [25]. The differences in the most common bleeding source could be due to differences in patient/clinical characteristics. The average age of patients among whom tumors were the most common [24] was low at 48.2 years, and DBE alone was used in that reported series. Vascular lesions were found at a fairly low rate in our series, but this could be because nonspecific red spots were considered unlikely sources of bleeding and were excluded from among the vascular lesions identified in our patients. A small angioectasia can be a source of bleeding that is easily overlooked during CE. In recent years, flexible spectral imaging color enhancement (FICE) has been added to RAPID6.5 as an image enhancement mode [40], and we have reported the usefulness of CE with FICE for visualizing small-bowel lesions such as angioectasia, erosion/ulceration, and various tumors [41]; FICE improves detection of angioectasia [42]. We anticipate future refinement of FICE-based diagnostic strategies for minute angioectasia.

Not all factors associated with positive CE findings in cases of occult OGIB are clear. However, in this study, it was shown that small-bowel lesions should be suspected in patients with occult OGIB and a high platelet count. If analyses of larger patient groups can establish predictor variables for various small-bowel lesions such as ulcers, vascular lesions, tumors, and other types of lesions, a standard diagnostic strategy that includes small-bowel testing can also be established.

## 5. Conclusion

With respect to detection of small-bowel lesions and identification of the various types of lesions, we found no difference in the diagnostic yield of CE between overt and occult OGIB cases. We recommend that CE be performed as actively for patients with occult OGIB as for those with overt OGIB.

## Figures and Tables

**Table 1:** Characteristics of patients with OGIB, per study group.

Characteristic	Occult OGIB	Overt OGIB	*P* value
	(*n* = 102)	(*n* = 325)	
Sex ratio (male/female)	50/52	198/127	<0.05
Age (years)	65.2 ± 17.3	65.3 ± 15.8	NS
Mean time from the first OGIB episode (months)	2.8 ± 2.7	1.7 ± 2.7	NS
Mean hemoglobin concentration at time of examination (g/dL)	7.8 ± 2.1	8.2 ± 2.7	NS
Plasma ferritin (mg/L)	10.1 ± 8.9	137.8 ± 210	0.0003
Blood transfusion	16 (16%)	110 (34%)	<0.01

Number of patients or mean ± SD values are shown.

NS: not significant.

**Table 2:** Final diagnoses per study group.

	Occult OGIB	Overt OGIB	*P* value
	(*n* = 102)	(*n* = 325)	
Small-bowel lesion	33 (32)	106 (33)	NS
Vascular lesion	11 (11)	31 (10)	NS
Ulcer or erosive lesion	18 (18)	51 (16)	NS
Tumor	4 (3)	20 (6)	NS
Other	0	4 (1)	NS
No small-bowel lesion	69 (68)	219 (67)	NS

Number (%) of patients is shown.

NS: not significant.

**Table 3:** Cases of occult OGIB due to tumor.

Patient	Final diagnosis	Indication for CE	Treatment
26-year-old woman	Aberrant pancreas	Positive FOBT, IDA	Surgery
40-year-old woman	T-cell lymphoma	Positive FOBT, IDA	Chemotherapy
68-year-old woman	Jejunum carcinoma	Positive FOBT, IDA	Surgery
81-year-old woman	GIST	IDA	Surgery

FOBT: fecal occult blood test; IDA: iron deficiency anemia; GIST: gastrointestinal stromal tumor.

**Table 4:** Diagnosis (final) and treatment modalities, per bleeding type.

Final diagnosis	Diagnostic modality	Treatment modality
Occult OGIB (*n* = 33)		
Vascular lesion (11)	DBE (7), CE (4)	Endoscopic hemostasis (6), follow up (no treatment) (5)
Ulcer or erosive lesion (18)	CE (8), DBE (6), CE+ response to medical treatment (3), and biopsy by DBE (1)	Medication (6), withdrawal of NAIDs (1), and follow up (no treatment) (11)
Tumor (4)	Surgery (3), biopsy by DBE (1)	Surgery (3), medication (1)^†^
Overt OGIB (*n* = 106)		
Vascular lesion (31)	DBE (29), CE (2)	Endoscopic hemostasis (26), IVR (1), and follow up (no treatment) (4)
Ulcer or erosive lesion (51)	DBE (33), CE (15), biopsy by DBE (2), CE+ response to medical treatment (1)	Medication (18)^†^, withdrawal of NSAIDs (9), surgery (2), endoscopic hemostasis (1), and follow up (no treatment) (21)
Tumor (20)	Surgery (12), resection by DBE (4), CE (3), and biopsy by DBE (1)	Surgery (12), endoscopic resection (4), medication (1)^†^, and follow up (no treatment) (3)
Other (4)	DBE (3), CE (1)	Endoscopic hemostasis (1), follow up (no treatment) (3)

Number of patients is shown.

^†^Anti-tubercular drugs, 5-ASA, steroid, medication for gastritis, or chemotherapy.

CE: capsule endoscopy; DBE: double balloon endoscopy; OGIB: obscure gastrointestinal bleeding; IVR: interventional radiology; NSAIDs: non-steroidal anti inflammatory drugs.

**Table 5:** Clinical characteristics of patients with occult OGIB, per diagnosis.

Characteristic	Vascular lesion	Ulcer or erosive lesion	Tumor	No lesion
	(*n* = 11)	(*n* = 18)	(*n* = 4)	(*n* = 69)
Age (years)	68.8 ± 17.9	70.8 ± 16.1	53.8 ± 25.2	63.4 ± 17.5
Sex ratio (male/female)	9/2^a^	8/10	0/4	33/36
Laboratory values				
WBC (/*μ*L)	5287 ± 1553	6030 ± 2416	5958 ± 1402	5287 ± 2130
Hemoglobin (g/dL)	7.4 ± 2.0	9.3 ± 2.4^b^	8.0 ± 2.4	7.6 ± 2.0
Platelets (/*μ*L)	21.5 ± 5.9	22.5 ± 8.7	33.6 ± 8.8^a^	23.0 ± 9.2
Total protein (g/dL)	6.6 ± 0.9	6.6 ± 0.6	6.1 ± 1.6	6.9 ± 0.6
Albumin (g/dL)	3.8 ± 0.5	3.9 ± 0.5	3.9 ± 0.3	4.0 ± 0.6
CRP (mg/dL)	0.40 ± 0.85	0.68 ± 0.96	0.08 ± 0.39	0.35 ± 0.80
Plasma ferritin (mg/L)	7.5 ± 4.6	13.5 ± 12.1	13.6 ± 11.0	9.3 ± 8.7
Symptom(s)				
Diarrhea	0 (0)	0 (0)	0 (0)	5 (7)
Body weight loss	0 (0)	0 (0)	1 (25)	2 (3)

Number (%) of patients or mean ± SD values is shown.

^a^ *P* < 0.05, ^b^ *P* < 0.01, compared with no lesion.

WBC: white blood cell count; CRP: C-reactive protein.
